# Supplementary material for: Predictors of Loss of Functional Independence in Parkinson’s Disease: Results from the COPPADIS Cohort at 2-Year Follow-Up and Comparison with a Control Group
Source: Diagnostics (Basel). 2021 Sep 29;11(10):1801. doi: 10.3390/diagnostics11101801 (PMC8534700; doi:10.3390/diagnostics11101801)
Supplement: Supplementary file 1 [file diagnostics-11-01801-s001.zip › diagnostics-1369599-supplementary.pdf]

Table S1 – Supplementary Material. Changes in disability (Schwab & England Activities of Daily Living Scale [S&E-ADLS] score) in PD patients and/or controls from V0 (baseline) to V2 (2 years ± 1 month). The change between both visits in both groups in other variables is shown as well.

	**PD patients V0**	**PD patients V2**	**Cohen´s test**	p_a	**Controls V0**	**Controls V2**	p^b	p^c	p^d
S&E-ADLS score	88.58 ± 10.19	84.26 ± 13.38	-0.519	<0.0001	98.87 ± 6.52	99.52 ± 2.15	0.227	0.069	<0.0001
Hoehn & Yahr (OFF)	1.93 ± 0.53	2.1 ± 0.52	0.422	<0.0001	N. A.	N. A.	N. A.	N. A.	N. A.
UPDRS-III (OFF)	21.92 ± 10.53	25.26 ± 12.19	0.452	<0.0001	N. A.	N. A.	N. A.	N. A.	N. A.
UPDRS-IV	1.99 ± 2.41	2.65 ± 2.75	0.371	<0.0001	N. A.	N. A.	N. A.	N. A.	N. A.
FOGQ	3.76 ± 4.69	4.94 ± 5.18	0.400	<0.0001	N. A.	N. A.	N. A.	N. A.	N. A.
LEDD (mg)	577.48 ± 412.09	767.56 ± 307.1	0.812	<0.0001	N. A.	N. A.	N. A.	N. A.	N. A.
Number of non-antipark. drugs	2.35 ± 2.38	3.08 ± 2.65	0.507	<0.0001	2.04 ± .2.16	2.76 ± 2.35	0.002	0.049	N. A.
PD-CRS	92 ± 15.65	90.26 ± 18.07	-0.207	<0.0001	99.65 ± 13.56	99.68 ± 13.73	0.375	0.912	0.246
NMSS	45.08 ± 37.62	53.55 ± 42.28	0.343	<0.0001	14.74 ± 18.72	14.65 ± 21.82	0.402	0.923	0.159
BDI-II	8.28 ± 6.9	8.54 ± 7.48	0.048	0.375	4.56 ± 5.46	4.31 ± 5.5	0.481	0.610	0.536
PDSS	117.13 ± 24.48	117.85 ± 24.98	0.038	0.430	131.26 ± 17.41	126.67 ± 26.46	0.776	0.425	0.632
QUIP-RS	4.6 ± 8.8	4.66 ± 9.22	0.007	0.798	1.51 ± 3.73	1.32 ± 3.37	0.163	0.564	0.806
NPI	5.82 ± 7.88	6.17 ± 9.39	0.056	0.484	3.31 ± 7.15	2.64 ± 7.67	0.756	0.697	0.065
VAS-PAIN	2.61 ± 2.92	2.96 ± 2.88	0.147	0.036	1.49 ± 2.41	1.70 ± 2.32	0.269	0.840	0.883
VASF − physical	2.86 ± 2.67	3.17 ± 2.8	0.147	0.018	1.52 ± 2.35	1.29 ± 2.12	0.780	0.221	0.717
VASF − mental	2.09 ± 2.51	2.20 ± 2.61	0.056	0.493	1.29 ± 2.09	1.03 ± 1.97	0.699	0.869	0.920
PDQ-39SI	16.72 ± 13.02	20.3 ± 16.41	0.413	<0.0001	N. A.	N. A.	N. A.	N. A.	N. A.
PQ-10	7.28 ± 1.53	7.13 ± 1.52	-0.114	0.048	8.07 ± 1.22	7.86 ± 1.65	0.050	0.489	0.207
EUROHIS-QOL8	3.8 ± 0.7	3.68 ± 0.67	0.048	0.387	4.18 ± 0.5	4.12 ± 0.51	0.065	0.954	0.291

P values were computed using general linear models (GLM) repeated measures. The results represent mean ± SD or %; p_a, change over time (V2 vs V0) in PD patients; p_b, change over time (V2 vs V0) in controls. Age, gender, and LEDD (levodopa equivalent daily dose) (except for assessing changes in this variable) at V0 and at V2 were included as covariates for disability analysis; for the rest of variables, GLM measures were adjusted also to S&E-ADLS score at V0 and to the change in S&E-ADLS score from V0 to V2; p^c, group visit interaction; p^d, PD vs controls. PD vs controls is not applicable if test of interaction was significant (a significant test of interaction means the rates of changes over time are different between the two groups). All patients with the data at V0 and V2 were included for each comparative analysis. For S&E-ADLS, N=507 in PD patients and N=124 in the control group.

ADLS, Schwab & England Activities of Daily Living Scale; BDI-II, Beck Depression Inventory-II; FOGQ, Freezing Of Gait Questionnaire; NMSS, Non-Motor Symptoms Scale; NPI, Neuropsychiatric Inventory; PD-CRS, Parkinson’s Disease Cognitive Rating Scale; PDSS, Parkinson’s Disease Sleep Scale; QUIP-RS, Questionnaire for Impulsive-Compulsive Disorders in Parkinson’s Disease-Rating Scale; UPDRS, Unified Parkinson’s Disease Rating Scale; VAFS, Visual Analog Fatigue Scale; VAS-Pain, Visual Analog Scale-Pain.
